# Serum CCL20 combined with IL-17A as early diagnostic and prognostic biomarkers for human colorectal cancer

**DOI:** 10.1186/s12967-019-2008-y

**Published:** 2019-08-06

**Authors:** Dan Wang, Weitang Yuan, Yaping Wang, Qian Wu, Li Yang, Feng Li, Xinfeng Chen, Zhen Zhang, Weina Yu, Nomathamsanqa Resegofetse Maimela, Ling Cao, Dong Wang, Junxia Wang, Zhenqiang Sun, Jinbo Liu, Yi Zhang

**Affiliations:** 1 Biotherapy Center, The First Affiliated Hospital of Zhengzhou University, Zhengzhou, 450052 Henan People’s Republic of China; 2 Cancer Center, The First Affiliated Hospital of Zhengzhou University, Zhengzhou, 450052 Henan China; 3 Department of Anorectal Surgery, The First Affiliated Hospital of Zhengzhou University, Zhengzhou, 450052 Henan China; 4 Department of Gastrointestinal Surgery, The First Affiliated Hospital of Zhengzhou University, Zhengzhou, 450052 Henan China; 5 School of Life Sciences, Zhengzhou University, Zhengzhou, 450052 Henan China; 6 Henan Key Laboratory for Tumor Immunology and Biotherapy, Zhengzhou, 450052 Henan China

**Keywords:** Colorectal cancer, Diagnosis, Prognosis, CCL20, IL-17A

## Abstract

**Background:**

Noninvasive and effective methods of early diagnosis of colorectal cancer (CRC) are underexplored. Inflammation is known to play an important role in the tumor microenvironment of CRC. Therefore, the aim of this study was to elucidate novel inflammatory biomarkers related to early diagnosis and prognosis of CRC.

**Methods:**

Based on the results from a multiplex assay and a pan-cancer screening of TCGA data with 18 cancer types, we identified several targeted biomarkers. We further confirmed these results using a trial cohort of 112 CRC patients and 151 controls (59 healthy donors, 52 colitis and 40 colorectal adenoma patients) by Elisa and immunohistochemistry (IHC). The biomarkers expression levels in CRC patients of different clinical stages were compared. The targeted biomarkers panel was developed using logistic regression model and was then validated using an independent cohort including 75 CRC patients and 90 controls (35 healthy donors, 20 colitis and 35 colorectal adenoma patients). Diagnostic accuracy was evaluated using area under the receiver-operating characteristic (ROC) curve and overall survival analysis was used for prognosis. Gene ontology (GO) analyses and Gene set enrichment analyses (GSEA) were performed to predict the function of the candidate biomarkers.

**Results:**

CCL20 and IL-17A were identified as candidate biomarkers using multiplex assay and pan-cancer screening of TCGA data. Elisa and IHC demonstrated that both CCL20 and IL-17A levels were highly expressed in CRC patients, more especially in patients with advanced stage disease. A signature expression of the two biomarkers showed high diagnostic accuracy of CRC. Importantly, the diagnostic sensitivity and specificity were still satisfactory in the early stage and low carcinoembryonic antigen (CEA) level groups. Bioinformatics analysis revealed that CCL20 and IL-17A may be involved in CRC progression. In addition, the diagnostic performance of CCL20 and IL-17A in combination was superior to that of either marker alone.

**Conclusions:**

Serum CCL20 and IL-17A levels were identified as independent prognostic markers for CRC. The CCL20-IL-17A panel exhibited a good performance in the diagnosis of early stage CRC.

**Electronic supplementary material:**

The online version of this article (10.1186/s12967-019-2008-y) contains supplementary material, which is available to authorized users.

## Background

Colorectal cancer (CRC) is one of the most common cancers, and the second leading cause of cancer related mortality globally [1]. Reports indicate that the 5-year relative survival for CRC patients ranges from greater than 90% (in early stage patients) to slightly greater than 10% (in stage IV patients) [2]. Therefore, screening for early stage CRC biomarkers is crucial for the prevention and proper management of disease progression and for controlling CRC-induced mortality [3]. Despite the development of screening strategies such as colonoscopy, fecal occult-blood testing, and stool DNA test, early diagnosis of CRC remains unrealized. Recently, the investigation of noninvasive biomarkers has become a hotspot. However, the frequently used serum tumor biomarkers carcinoembryonic antigens (CEA) and carbohydrate antigens 19-9 (CA19-9), present with limited sensitivity and specificity to early stage CRC diagnosis and yield controversial prognostic values [4, 5]. Therefore, the identification of some novel and noninvasive biomarkers for early diagnosis of CRC are extremely high in demand.

Cancer-associated inflammation has been identified as a key determining factor of disease progression and survival in CRC patients. A good number of studies have associated CRC patients with high incidences of inflammatory bowel disease. Chemokines and cytokines are essential inflammatory factors for malignant tumor development. Chemokines can be classified into 4 main families: CC, CXC, CX3C, and C. CXC and CC are the largest families and are involved in regulating leukocyte trafficking and tumor cell biological properties. Chemokine CC ligand 20 (CCL20) is the only chemokine that interacts with the G-protein coupled 7-transmembrane receptor CCR6. Previous studies indicate that CCL20 has the ability to recruit lymphocytes and has been correlated with tumor progression in various malignant neoplasms [6, 7]. Moreover, Chang et al., previously described CCL20 as a novel serum marker for detecting nasopharyngeal carcinoma [8]. However, the diagnostic and prognostic values of serum CCL20 in CRC patients have not been fully evaluated.

Interleukins are equally an important group of cytokines. They have been strongly associated with inflammatory responses in the tumor microenvironment. Interleukin (IL)-17A, referred to as IL-17, is secreted by a subset of activated CD4^+^ T cells (Th17), some CD8^+^ T cells, and γδ T cells [9, 10]. Study reports indicate that IL-17A is responsible for the aggressive progression of several malignant tumors [11, 12]. Likewise, the IL-17A level in tissues have been shown to play a major role in CRC progression [13, 14]. In addition, high baseline serum IL-17A concentrations have been associated with shorter progression-free survival in CRC patients [15], and tumor dissemination in small cell lung cancer patients [16]. Thus far, the potential role of serum IL-17A in identifying patients with CRC has not been investigated.

IL-17A has also been shown to enhance CCL20 production in various tumor cells [17, 18]. CCL20 preferentially mediates the recruitment of Th17 cells to tumor tissues, resulting in increased IL-17A secretion [19]. Therefore, CCL20 may be closely associated with IL-17A. Nevertheless, the role of both serum chemokines and cytokines in diagnosing or predicting long-term outcomes in CRC patients has not been fully evaluated. In this study, we investigated the expression levels of CCL20 and IL-17A in CRC patients and elucidated the effects of their serum levels on disease status. More importantly, we sought to establish a highly specific and sensitive CCL20-IL-17A-based model for evaluating early stage CRC patients and assessed the prognostic value for CRC patients.

## Materials and methods

### Study design

**Fig. 1:**
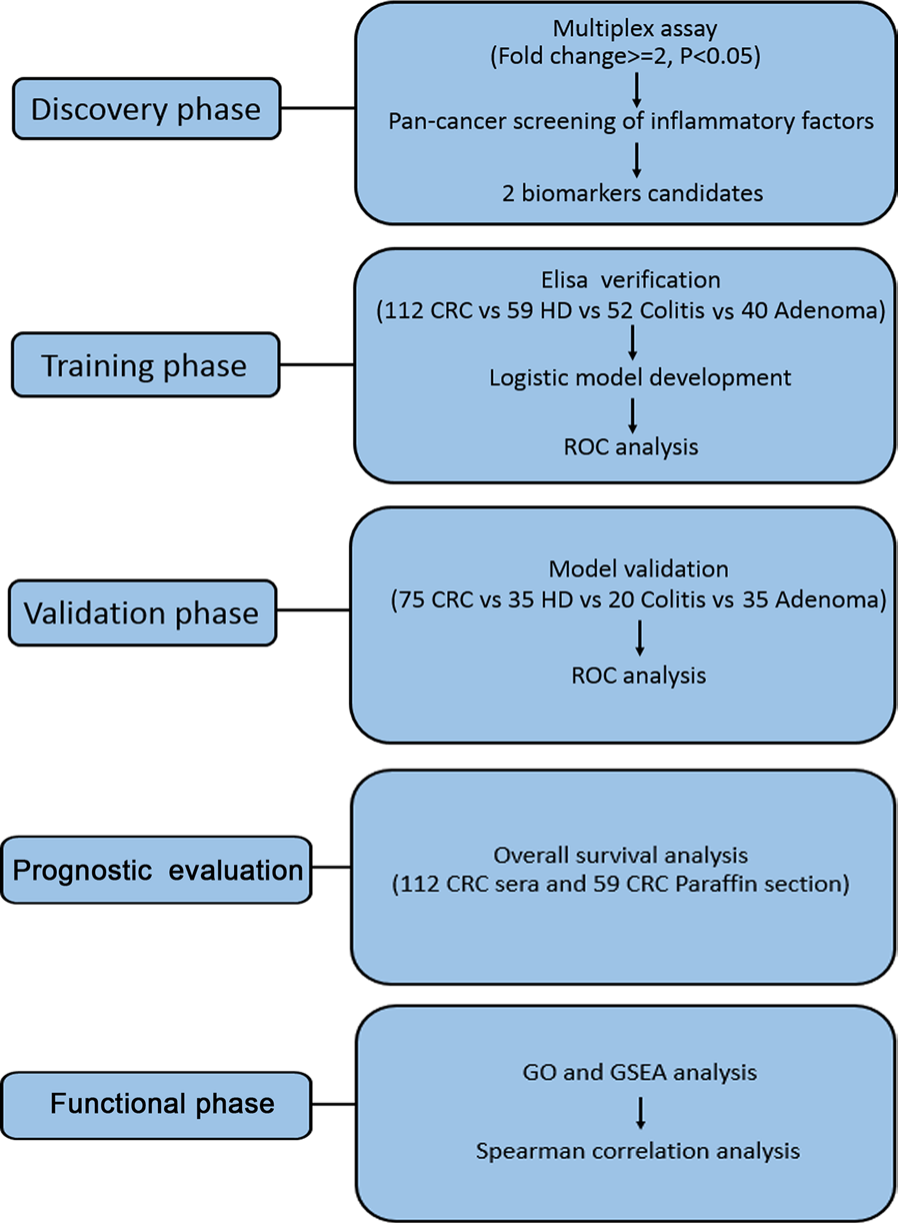
Design of the study (CRC, colorectal cancer; HD, healthy donor)

The whole study consisted of five phases: the discovery phase, training phase, validation phase, prognostic evaluation and functional phase (Fig. 1). In the discovery phase, serum samples were collected from 3 CRC patients and 3 healthy controls. We conducted a multiplex assay on each of the samples to identify the potential biomarkers. We then proceeded to conduct a pan-cancer screening of inflammatory factors (CCL/CXCL chemokines and interleukins) using data from 18 cancer types from TCGA and identified CCL20 and IL-17A as biomarker candidates. We confirmed the identified targeted biomarkers in the training phase by making use of an independent training cohort of 112 CRC patients and 151 controls (59 HD, 52 colitis and 40 colorectal adenoma patients) by Elisa and immunohistochemistry (IHC). The logistic regression model was adopted to develop the panel of the targeted biomarkers. To further evaluate the diagnostic accuracy of the model, we used receiver-operating characteristic (ROC) curves. Subsequently, in the validation phase, the performance of the logistic model was validated by differentiating CRC patients from the control group in another independent validation cohort including 75 CRC patients and 35 healthy controls, 20 colitis and 35 colorectal adenoma patients.

In the prognostic evaluation phase we evaluated the value of the targeted markers, conducting overall survival analysis using independent samples from the training cohort of 112 CRC serum and 59 tumor samples from CRC patients. Finally, in the functional phase, we used Gene ontology (GO) analyses and Gene set enrichment analyses (GSEA) to evaluate the function of the candidate genes. TCGA database were used to assess the correlation between the candidate genes and the genes related to cancer progression.

### Clinical sample selection

We collected serum samples of healthy controls, and untreated colitis, colorectal adenoma, and CRC patients. The blood samples were obtained from the Departments of Oncology and Gastroenterology of the First Affiliated Hospital of Zhengzhou University from January 2013 to December 2015. The healthy controls, colitis, and colorectal adenoma patients were enrolled from the Department of Physical Examination Center and Clinical Laboratory of the First Affiliated Hospital of Zhengzhou University. The paraffin section of tissue samples from CRC patients (n = 59) diagnosed between 2011 and 2013 were obtained from the Pathology Department. Patients were classified according to UICC-TNM classification and were graded according to the WHO classification criteria as well, moderately, or poorly differentiated. All CRC patients were histologically diagnosed. Age- and sex-matched controls were selected (Additional file 1: Table S1). Serum was transferred to 1.5 ml tubes and stored at − 80 °C for further processing. Protocols for this study were approved by the local ethics committee (Ethical approval number: Science-2010-LW-1213), and an informed consent was obtained from each patient and volunteer, with available follow-up information.

### Database

Genes with higher expression in CRC tumor tissue, were screened by The Cancer Genome Atlas (TCGA) database (http://www.cancaergenome.nih.gov), which provided data of 18 cancer types. Moreover, the CRC transcriptional expression data (GDS4718) were downloaded from the Gene Expression Omnibus (GEO) database for the correlation analyses. It contained 19 normal colon mucosa (N) and 19 primary tumor (Ca) samples.

### Multiplex assay

To identify which factor was highly expressed in CRC patients compared to healthy donors, a multiplex assay was used. The levels of cytokines and chemokines in sera were analyzed using a multi-analyze flow assay kit (BioLegend, USA), including 13 human cytokines (Cat. 740001) and 13 human chemokines (Cat. 740003), according to the manufacturer’s instructions.

### Elisa

Cytokine levels were measured using the highly sensitive Elisa kits (R&D Systems, Minneapolis, MN, USA), specific for human cytokines, according to the manufacturer’s instructions. Detailed methods are described in Additional file 2: Methods section.

### Immunohistochemistry

Immunohistochemistry was performed as previously described [20]. Mouse anti-CCL20 and rabbit anti-human IL-17A (1:300; Abcam, USA) were used as primary antibodies. Detailed methods are described in Additional file 2: Methods section.

### Statistical analyses

All statistical analyses were performed using the SPSS 17.0 software. Data of different groups were compared using the Student’s *t* test, Chi square test, and one-way ANOVA. Kaplan–Meier curves analysis, spearman correlation analysis, and univariate and multivariate logistic regression models were performed. A diagnostic model was developed using logistic regression analysis. ROC curves were constructed by plotting sensitivity vs. specificity, and the areas under the curves (AUC) were analyzed with the Hanley and McNeil method. A p value < 0.05 was considered statistically significant (*p < 0.05, **p < 0.01, ***p < 0.001).

## Results

### Identification of candidate biomarkers for CRC

**Fig. 2:**
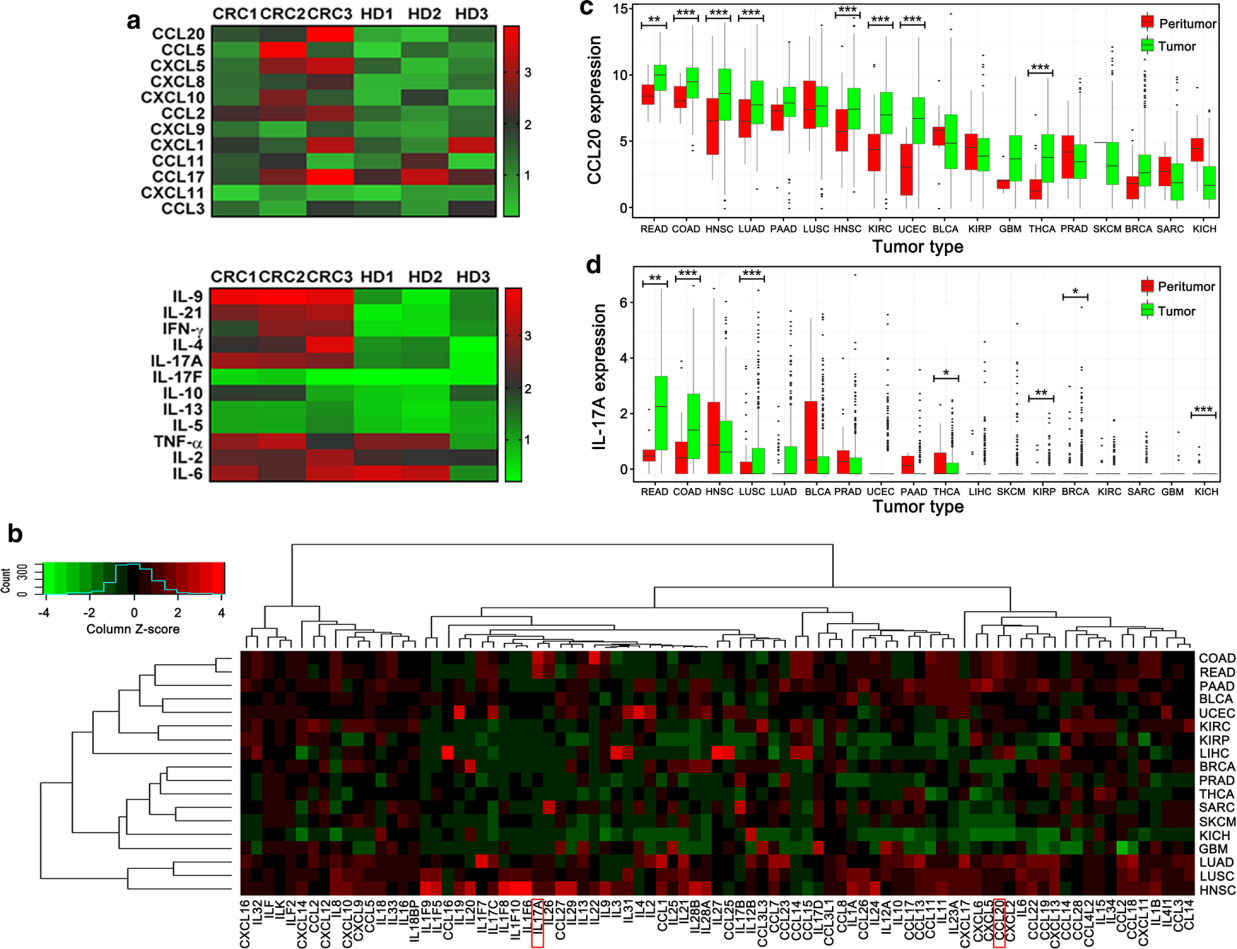
CCL20 and IL-17A are highly expressed in tumor and sera of CRC patients. **a** The expression level of cytokines and chemokines in sera from patients with CRC and HD were analyzed using a multiplex assay kit. **b** Hierarchical clustering analysis of differentially expressed genes of CCL/CXCL families and interleukins families download from TCGA database in 18 cancer types. **c**, **d** The expression of CCL20 and IL-17A in 18 kinds of cancer tissues and the corresponding normal tissues (*p < 0.05; **p < 0.01; ***p < 0.001)

To identify potential CRC biomarkers, we examined the levels of serum inflammatory cytokines and chemokines in CRC patients and HD using a multiplex assay. The serum levels of CCL20, CXCL10, CCL2, IL-9, IL-21, and IL-17A were significantly higher in CRC patients than in HD (fold change ≥ 2, p < 0.05) (Fig. 2a). To determine the highly expressed genes in CRC patients, pan-cancer screening of CCL/CXCL chemokine and interleukin families was performed for tumor tissues from 18 cancer types via TCGA data. We observed that CCL20 and IL-17A expression in colon adenocarcinoma (COAD) and rectal adenocarcinoma (READ) tissues were higher than the expression in other cancer tissues (Fig. 2b and Additional file 3: Fig. S1A). After comparing expression levels between cancer tissues and the corresponding normal tissues, we observed that the levels of both markers were higher in CRC cancer tissues than in normal tissues (Fig. 2c, d). After superimposing results from Fig. 2a, b, we realized that CCL20 and IL-17A may exhibit good performances in predicting the diagnosis and prognosis of CRC patients. As a result, CCL20 and IL-17A levels were identified as candidate CRC biomarkers.

### Confirmation of CCL20 and IL-17A levels by Elisa and IHC

**Fig. 3:**
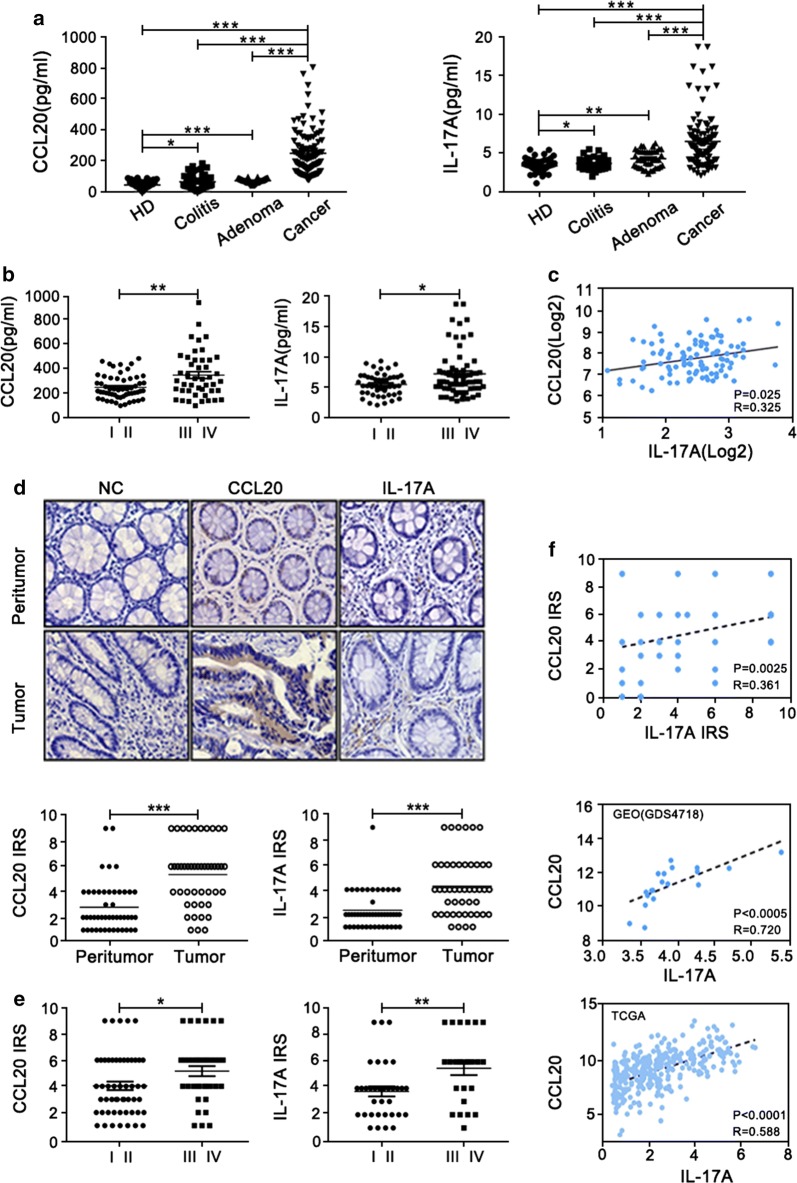
Expression of CCL20 and IL-17A were higher and closely related in sera and tumor tissues of CRC patients. **a** Evaluation of the expression of CCL20 and IL-17A in sera from 112 CRC patients, 59 HD, 52 colitis and 40 colorectal adenoma patients. Student’s t test was used. **b** The expression of serum CCL20 and IL-17A in different stage. Student’s t test was used. **c** Correlation between serum levels of CCL20 and IL-17A in CRC patients. Spearman correlation coefficient was calculated. **d** Immunohistochemical staining of CCL20 and IL-17A in paired adjacent normal and tumor tissues from one case. Immune reactivity score (IRS) was evaluated in paired adjacent normal and tumor tissues. **e** IRS was evaluated in different stage. **f** Correlation between protein levels of CCL20 and IL-17A in tumor tissues from CRC patients and correlation between mRNA levels of CCL20 and IL-17A from GEO and TCGA databases (*p < 0.05; **p < 0.01; ***p < 0.001)

To verify the serum levels of CCL20 and IL-17A in CRC patients, 112 samples from initially treated CRC patients and 151 controls (59 HD, 52 colitis, 40 colorectal adenoma patients) were collected. There were no significant differences in the age and sex of the groups in this cohort (Additional file 1: Table S1). As shown in Fig. 3a, serum CCL20 and IL-17A levels in CRC patients were significantly higher than those in the controls (p < 0.0001). The two markers expression in CRC patients were also higher than that in other cancer patients (Additional file 3: Fig. S1B). Moreover, CCL20 and IL-17A levels were elevated in CRC patients with advanced stages (p < 0.01, p < 0.05; Fig. 3b). Spearman correlation analysis indicated a significant positive correlation between these two biomarkers (r = 0.325, p = 0.025; Fig. 3c). Furthermore, we observed that the levels of the two biomarkers were significantly higher in tumor tissues when compared with the levels in the peritumoral tissues (p < 0.001; Fig. 3d). CCL20 was highly expressed in the cytoplasm of tumor cells, and IL-17A was mainly from infiltrating lymphocytes (Fig. 3d). CCL20 and IL-17A levels were also significantly higher in the advanced stage than in the early stage of CRC (p < 0.05, p < 0.01; Fig. 3e). A similar correlation analysis was performed, and a positive correlation was observed between the protein levels of CCL20 and IL-17A in tumor tissues (r = 0.361, p = 0.0025; Fig. 3f). This correlation was confirmed by the mRNA levels obtained from the GEO (r = 0.720, p < 0.0005; Fig. 3f) and TCGA (r = 0.588, p < 0.0001; Fig. 3f) databases. These data strongly confirmed that expression of CCL20 and IL-17A were higher in CRC patients.

### Relationship between serum CCL20 and IL-17A levels, and clinicopathological parameters

112 CRC samples were collected to evaluate the correlation of serum CCL20 and IL-17A levels with the clinicopathological characteristics of patients. As shown in Additional file 1: Tables S2, S3, higher expression of CCL20 and IL-17A together or respectively were significantly associated with higher T stage, N stage, M stage, overall stage, deep invasion, liver metastasis, larger tumor size, adenocarcinoma type, and CEA values. Our data suggested potential roles of CCL20 and IL-17A in CRC progression.

### Establishing the predictive model for CRC diagnosis

**Fig. 4:**
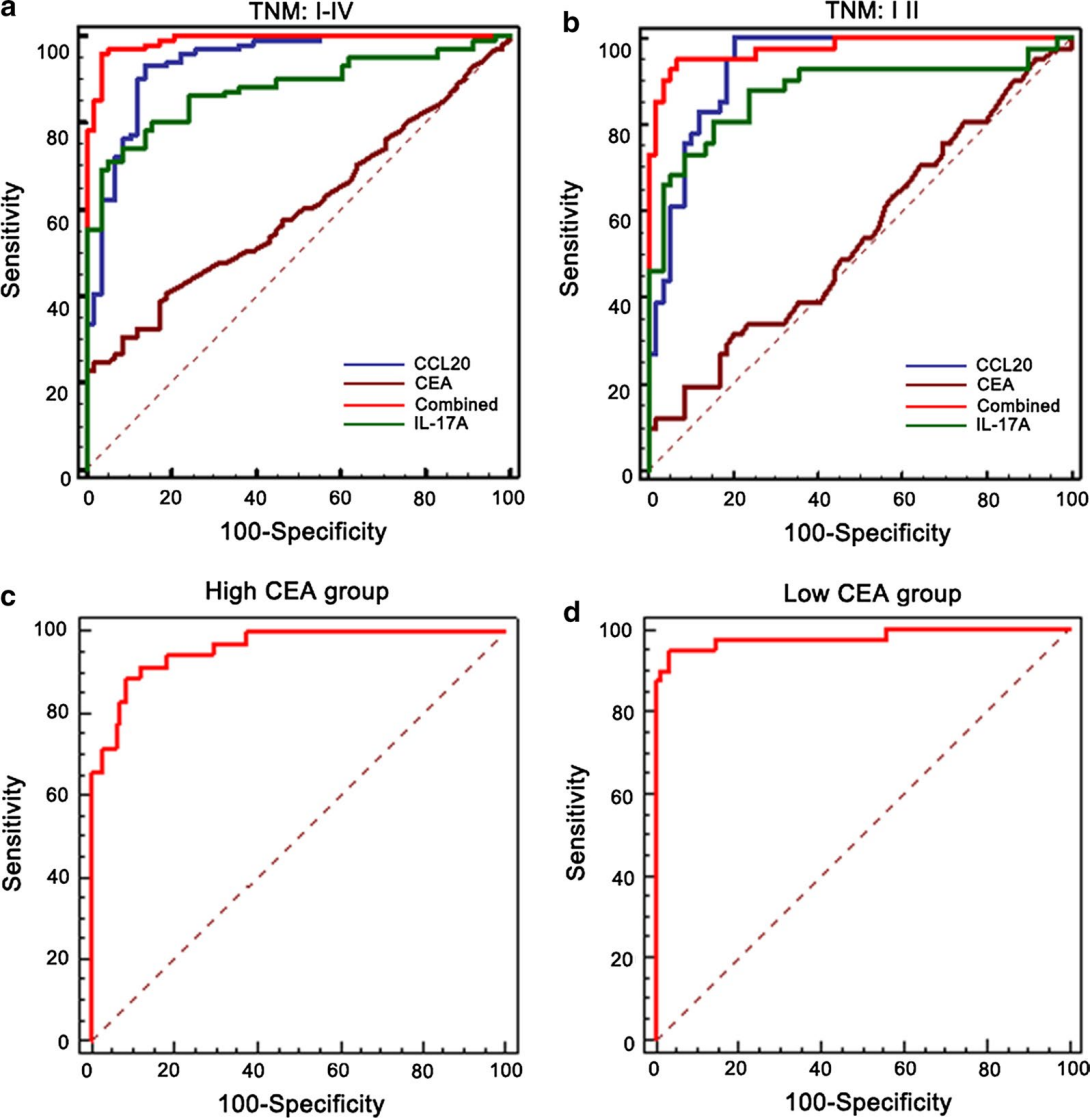
ROC curves for the ability of the CCL20-IL-17A panel to differentiate CRC patients from the control group in the training dataset. ROC curves of CCL20 and IL-17A for CRC cases with overall stages (I–IV) (**a**), early stages (I/II) (**b**), low-CEA level group (**c**) and high-CEA level group (**d**)

The efficacy of serum CCL20 and IL-17A in discriminating between CRC patients and controls was verified using the ROC curve. As shown in Fig. 4a, the efficacy of CCL20 only (AUC = 0.936; cut-off value: 89.64; sensitivity: 93.1%; specificity: 86.3%) or IL-17A only (AUC = 0.879; cut-off value: 4.7962; sensitivity: 24.8%; specificity: 98.3%) was better than that of CEA (AUC = 0.601; sensitivity: 24.8%; specificity: 98.3%). After combining CCL20 with IL-17A in a logistic regression model, the screening efficacy (AUC = 0.988; cut-off value: 0.54; positive value: 22.4%; negative predictive value: 95.9%; sensitivity: 96.1%; specificity: 96.5% for the combination; Fig. 4a) was better than their individual efficacies (Combination vs. CCL20: p = 0.015 and combination vs. IL-17A: p = 0.0001). The predicted probability of CRC diagnosis from the stepwise logistic regression model was calculated as follows:

$${\text{logit (P)}}\, = \, - 8. 8 2 2\, + \, 3. 7 4 3 {\text{ IL}} - 1 7 {\text{A}}\, + \,1.0 30{\text{ CCL2}}0.$$

Furthermore, we evaluated the diagnostic performance of the established panel in distinguishing CRC patients with early TNM stage from controls. Similar results were obtained in patients with early stages (I/II). The AUC values for CCL20 and IL-17A were 0.935 (cut-off value: 89.68; sensitivity: 97.1%; specificity: 79.7%) and 0.876 (cut-off value: 4.0969; sensitivity: 80.4%; specificity: 84.7%) respectively. The combination of the two biomarkers robustly increased the AUC to 0.976 (cut-off value: 0.1878; sensitivity: 95.1%; specificity: 93.2%). The differences between them were statistically significant (combination vs. CCL20: p = 0.0431; combination vs. IL-17A: p = 0.003) (Fig. 4b). Then, we evaluated the diagnostic accuracy of the established panel according to the CEA levels. In the high-CEA level (> 5 ng/ml) group, the AUC of the panel was 0.927 (sensitivity: 92.8%; specificity: 91.2%; Fig. 4c). In the low-CEA level (< 5 ng/ml) group, the AUC of the panel was 0.976 (sensitivity: 95.1%; specificity: 94.8%; Fig. 4d). Collectively, our data implied that serum levels of CCL20-IL-17A panels used as a screening protocol may improve the efficacy of early diagnosis in CRC patients.

### Validation of the panels in another independent dataset

**Fig. 5:**
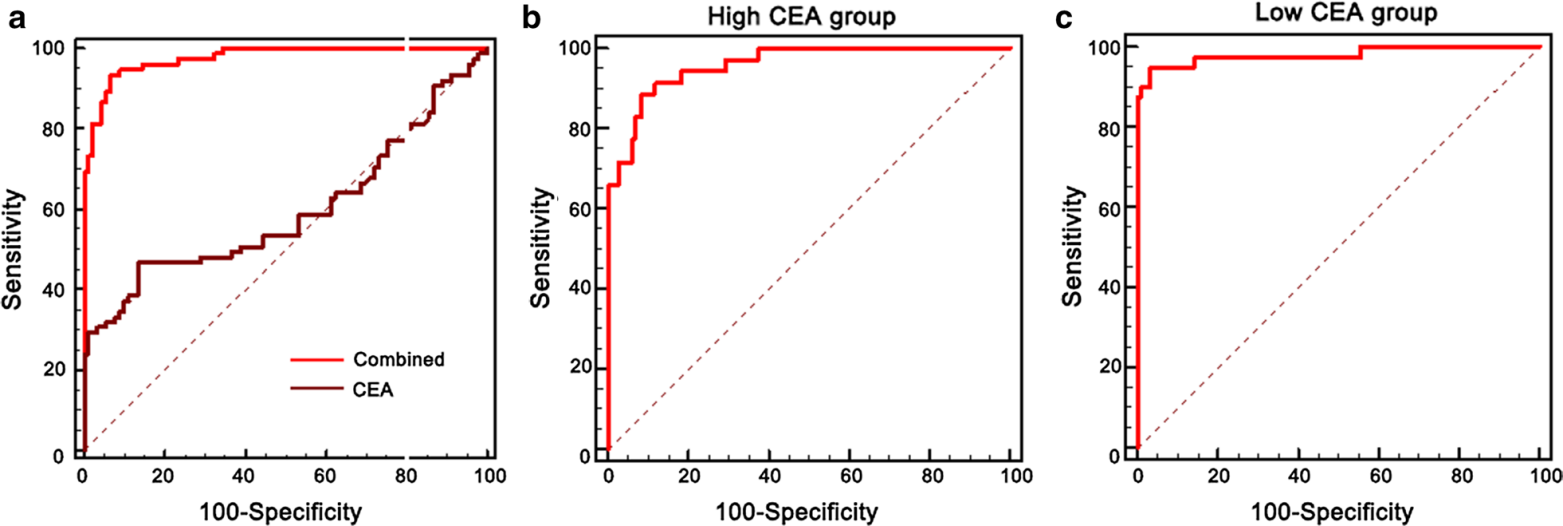
ROC curve analysis of the CCL20-IL-17A-based diagnostic model in distinguishing CRC cases with early stage from controls in the validation dataset. ROC curves of the model for CRC cases with early stage (**a**), low (**b**) and high (**c**) CEA level group

The model was validated in another cohort consisting of 75 patients with early stage CRC and 90 controls (35 HD, 20 colitis, and 35 colorectal adenoma patients) (Additional file 1: Table S1). As shown in Fig. 5a, the AUC value of the model (AUC = 0.976; sensitivity: 93.3%; specificity: 93.3%) was better than CEA (AUC = 0.596; sensitivity: 46.7%; specificity: 86.7%). Simultaneously, in the high-CEA level (> 5 ng/ml) group, the AUC of the panel was 0.959 (sensitivity: 88.6%; specificity: 91.7%; Fig. 5b). In the low-CEA level (< 5 ng/ml) group, the AUC of the panel was 0.981 (sensitivity: 95.0%; specificity: 96.7%; Fig. 5c). These results confirmed that the serum levels of CCL20-IL-17A panel-based screening protocol may improve the efficacy of early diagnosis in CRC patients.

### Prognosis evaluation

**Fig. 6:**
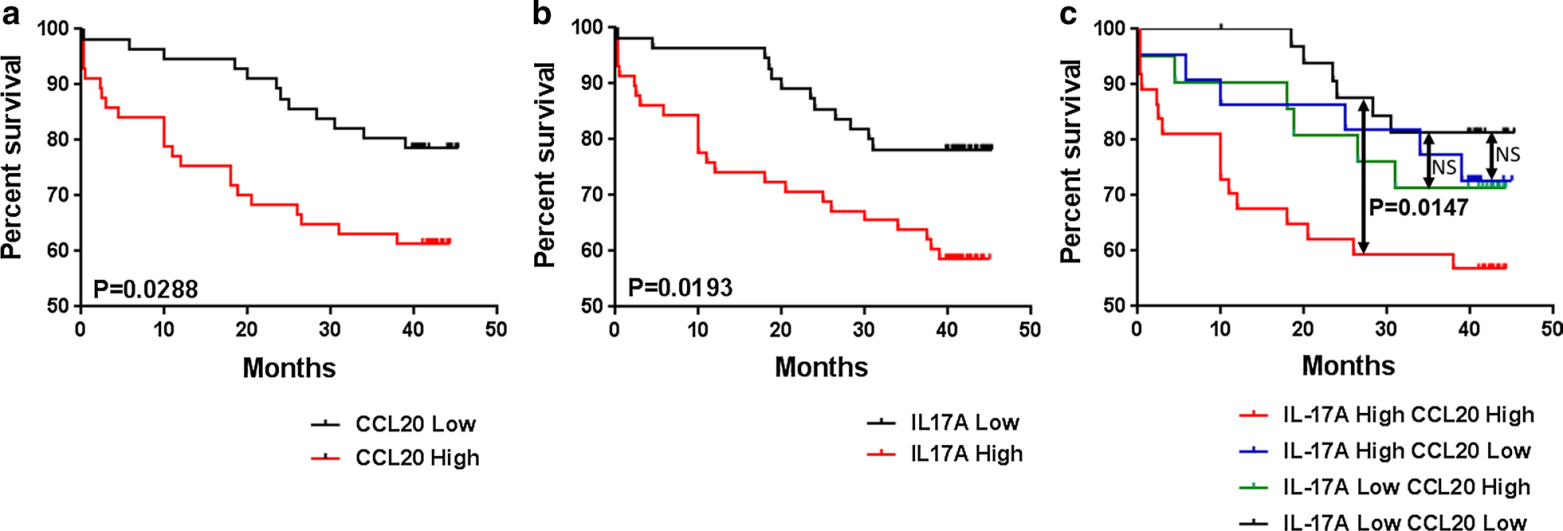
CCL20 and IL-17A levels were inversely associated with outcomes of CRC patients. **a**–**c** Serum CCL20 and IL-17A expression and follow-up data of 112 CRC patients were analyzed for the correlation between the two cytokines expression and survival

The overall survival (OS) times of CRC patients with lower serum level of CCL20 were longer compared with those with higher CCL20 expression (p = 0.0288, Fig. 6a). IL-17A expression levels were equally inversely associated with outcomes of CRC patients (p = 0.0193, Fig. 6b). Comparing to the patients with low expression of both biomarkers, patients with high expression of both biomarkers showed a evidently shorter OS (p = 0.0147), whereas patients with only one high-risk biomarker have no significant difference (Fig. 6c). It indicated that the two-biomarkers signature exhibited greater prognostic performance than one-single-biomarker.

As shown in Additional file 1: Table S4, univariate analysis revealed that N stage (hazard ratio = 2.60, p = 0.005), M stage (hazard ratio = 1.99, p = 0.03), liver metastasis (hazard ratio = 2.32, p = 0.04), age (hazard ratio = 1.97, p = 0.04), site of lesion (hazard ratio = 1.52, p = 0.04), overall stage (hazard ratio = 1.69, p = 0.02), serum CCL20 levels (hazard ratio = 2.32, p = 0.02), serum IL-17A levels (hazard ratio = 2.32, p = 0.014), and the combination of CCL20 and IL-17A (hazard ratio = 2.46, p = 0.03) were significantly associated with poor prognosis. Interestingly, a multivariate Cox’s regression analysis revealed that age (hazard ratio = 1.98, p = 0.04), site of lesion (hazard ratio = 3.14, p = 0.02), overall stage (hazard ratio = 1.37, p = 0.03), CCL20 (hazard ratio = 1.12, p = 0.001), IL-17A levels (hazard ratio = 1.22, p = 0.04) and the combination of CCL20 and IL-17A (hazard ratio = 1.45, p = 0.04) were independent prognostic factors for the OS of CRC patients (Additional file 1: Table S4). In summary, these results suggest significantly prognostic values of serum CCL20 and IL-17A levels in CRC patients.

### CCL20 and IL-17A function

**Fig. 7:**
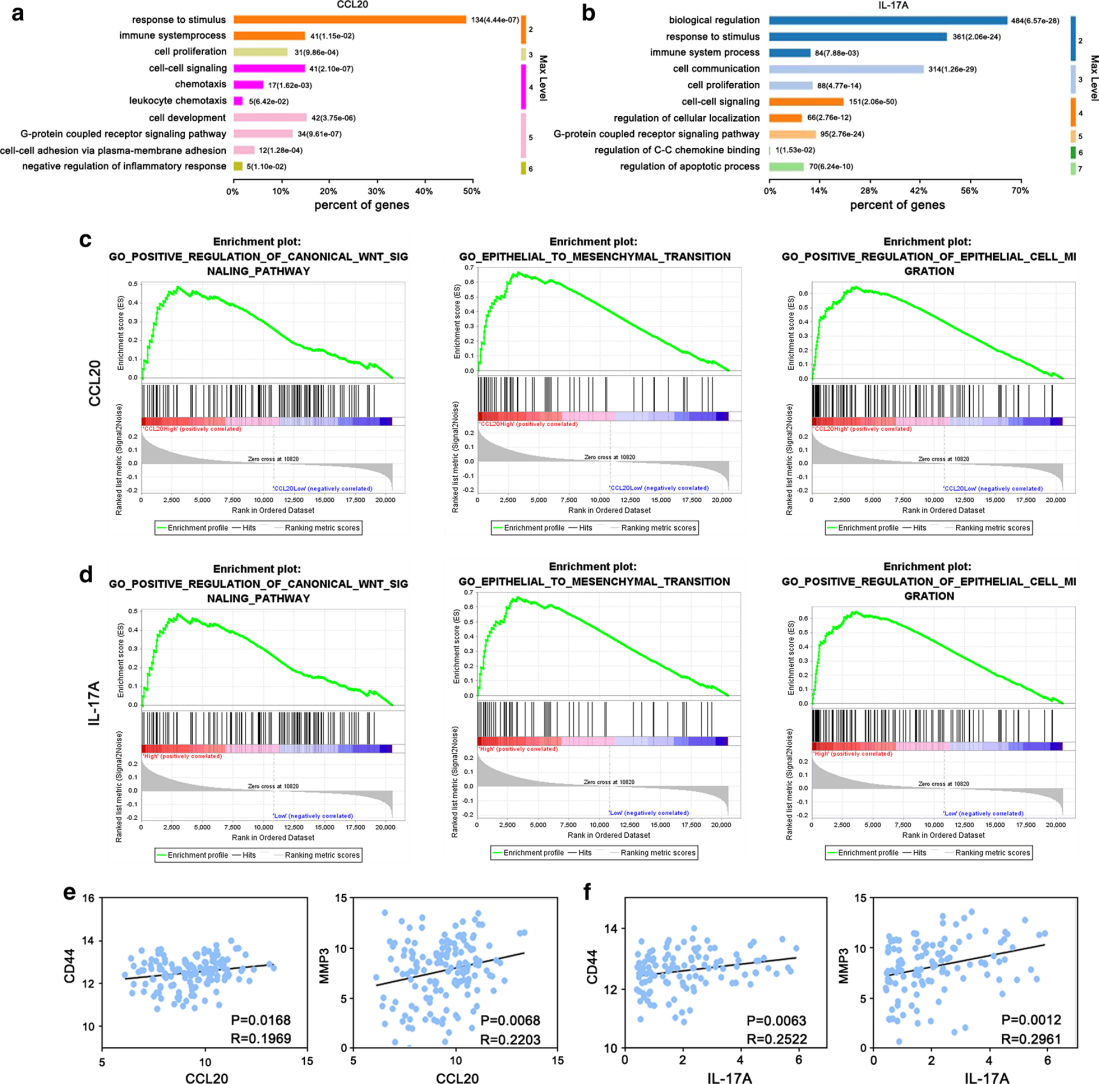
Functional analysis for CCL20 and IL-17A. **a**, **b** GO analysis of CCL20, IL-17A. **c**, **d** GSEA showed that CCL20, IL-17A were associated with positive regulation of canonical WNT signaling pathway, epithelial to mesenchymal transition (EMT), and positive regulation of epithelial cell migration. **e**, **f** Correlation between CCL20/IL-17A and CD44, MMP3 were analyzed. Spearman correlation coefficient was calculated

To analyze the function of CCL20 and IL-17A, differential expression genes were found according to the high and low expression groups indicated by the GOs. The main GO categories for differential expression genes were related to functions such as biological regulation, response to stimulus, cell–cell signaling, cell development, cell communication, and cell proliferation (Fig. 7a, b). Further analysis of GSEA, a powerful tool in inferring the biological function, was performed. The results indicated that genes that contributed to positive regulation of the canonical WNT signaling pathway, the epithelial to mesenchymal transition, and epithelial cell migration, were significantly enriched in both CCL20 highly expressed (Fig. 7c) and IL-17A highly expressed samples (Fig. 7d) of CRC patients. Finally, we observed that CCL20 and IL-17A levels were both positively associated with levels of CD44 and MMP3, which are closely related to cancer progression (Fig. 7e, f). Collectively, these results suggest that CCL20 and IL-17A levels play an important role in CRC development.

## Discussion

CRC is one of the leading causes of cancer mortality globally. Although there have been great advances in the diagnosis and prognosis of CRC in past decades, the dire need for improving early detection screening methods exist. Cytokines and chemokines have been recently reported to be of diagnostic and prognostic value in different cancers [21, 22]. There is a growing consensus that effective detection of early-stage cancer will likely rely on biomarker panels that have greater specificity and sensitivity, compared with single biomarkers [23, 24]. However, the potential diagnostic and prognostic roles of chemokine–cytokine combinations in serum have not been investigated. Therefore, this study aimed at identifying some potential biomarkers for early diagnosis and prognostic prediction of CRC.

In this study, we showed for the first time that a combination of serum CCL20 and IL-17A levels was a novel and effective diagnostic biomarker panel for early-stage CRC patients. Firstly, the expression of CCL20 and IL-17A in CRC tissues was significantly higher than that in other cancer tissues. Secondly, we found that the serum concentrations of CCL20 and IL-17A in CRC patients were significantly higher than those in healthy individuals. The higher expression of CCL20 and IL-17A was strongly correlated with advanced clinicopathological factors. Thirdly, the ROC curve analysis showed that a combination of CCL20 and IL-17A was an effective panel for discriminating CRC cases from HD, and colitis from colorectal adenoma cases. Surprisingly, this panel had the potential to distinguish stage I/II CRC patients from the controls. Finally, we also demonstrated that the panel was a more sensitive indicator than the conventional CEA biomarker. Even in the low-CEA level group, the diagnostic sensitivity and specificity of the panel remained satisfactory. Overall, we validated the high accuracy of a combination of CCL20 and IL-17A in the early diagnosis of CRC in patients.

This study equally revealed that CCL20 and IL-17A expression levels serve as prognostic biomarkers. Initially, we observed that the expression of CCL20 and IL-17A in the serum was closely associated with adverse clinicopathological parameters. In addition, their elevated serum levels were associated with poor survival. Moreover, serum CCL20 and IL-17A were observed as independent prognostic factors. Collectively, our study demonstrated that CCL20 and IL-17A may also be prognostic CRC predictors in addition to being potential diagnostic biomarkers.

Although serum CCL20 and IL-17A appear to be promising biomarkers, our current study is limited for several reasons. First, elevated CCL20 and IL-17A levels may be reflective of inflammatory conditions, which can affect the efficacy of disease detection. Another potential limitation of this study was the application of medians as the defined cut-off. Finally, clinical parameters are variable between institutions and/or individual clinicians; hence, the results of this small cohort may reflect biases inherent in the acquisition of such clinical data. Therefore, the validation of CCL20 and IL-17A as CRC biomarkers will require future large-scale counter screening.

## Conclusions

In summary, our study demonstrated that serum CCL20-IL-17A panels were effective diagnostic biomarkers for early-stage CRC patients. Moreover, high CCL20 and IL-17A levels predicted shorter OS in CRC patients and can be used as independent prognostic factors. In conclusion, our study provides the first evidence that serum CCL20 and IL-17A levels in combination may serve as highly effective early diagnostic biomarkers and prognostic predictors of CRC patients.

## Additional files

**Additional file 1.** Additional tables.

**Additional file 2.** Additional methods.

**Additional file 3: Fig. S1.** CCL20 and IL-17A expression are higher in CRC than the expression in other cancers. A. The expression of CCL20 and IL-17A from TCGA database in 18 kinds of cancer tissues. B. The levels of CCL20 and IL-17A in sera from CRC patients, lung adenocarcinoma [LUAD] patients, glioblastoma [GBM] patients and controls (Healthy donors [HD]) were tested by Elisa (*P < 0.05; **P < 0.01; ***P < 0.001).

## Data Availability

The datasets used and/or analyzed during the current study are available from the corresponding author on reasonable request.

## Abbreviations

CRC: colorectal cancer
HD: healthy donors
CEA: carcinoembryonic antigen
CCL20: chemokine CC ligand 20
N: normal colon mucosa
Ca: tumor
GO: gene ontology
GSEA: gene set enrichment analyses
GEO: Gene Expression Omnibus
TCGA: Cancer Genome Atlas
Elisa: enzyme-linked immune sorbent assay
IHC: immunohistochemistry
IRS: immune reactivity scores
AUC: areas under the curves
OS: overall survival
