# Correction to: Serum CCL20 combined with IL-17A as early diagnostic and prognostic biomarkers for human colorectal cancer

**DOI:** 10.1186/s12967-021-03107-7

**Published:** 2021-10-18

**Authors:** Dan Wang, Weitang Yuan, Yaping Wang, Qian Wu, Li Yang, Feng Li, Xinfeng Chen, Zhen Zhang, Weina Yu, Nomathamsanqa Resegofetse Maimela, Ling Cao, Dong Wang, Junxia Wang, Zhenqiang Sun, Jinbo Liu, Yi Zhang

**Affiliations:** 1 Biotherapy Center, The First Affiliated Hospital of Zhengzhou University, Zhengzhou, 450052 Henan People’s Republic of China; 2 Cancer Center, The First Affiliated Hospital of Zhengzhou University, Zhengzhou, 450052 Henan China; 3 Department of Anorectal Surgery, The First Affiliated Hospital of Zhengzhou University, Zhengzhou, 450052 Henan China; 4 Department of Gastrointestinal Surgery, The First Affiliated Hospital of Zhengzhou University, Zhengzhou, 450052 Henan China; 5 School of Life Sciences, Zhengzhou University, Zhengzhou, 450052 Henan China; 6 Henan Key Laboratory for Tumor Immunology and Biotherapy, Zhengzhou, 450052 Henan China

## Correction to: J Transl Med (2019) 17:253 10.1186/s12967-019-2008-y

The authors of the original article [1] have found out after publication that there were 2 figure errors in the article:

1. The ROC curves of the CCL20-IL-17A panel in Figure 4C and 4D were the same as that in the Figure 5B and 5C.
2. There is an image crack in the Figure 5A during a layer merging process.

These changes don’t affect any conclusions of the original paper.

The incorrect (Figs. 1 and 2) and correct figures (Figs. 3 and 4) are published in this correction article. The areas with the errors are indicated with a blue box. The original article has been updated.

**Fig. 1:**
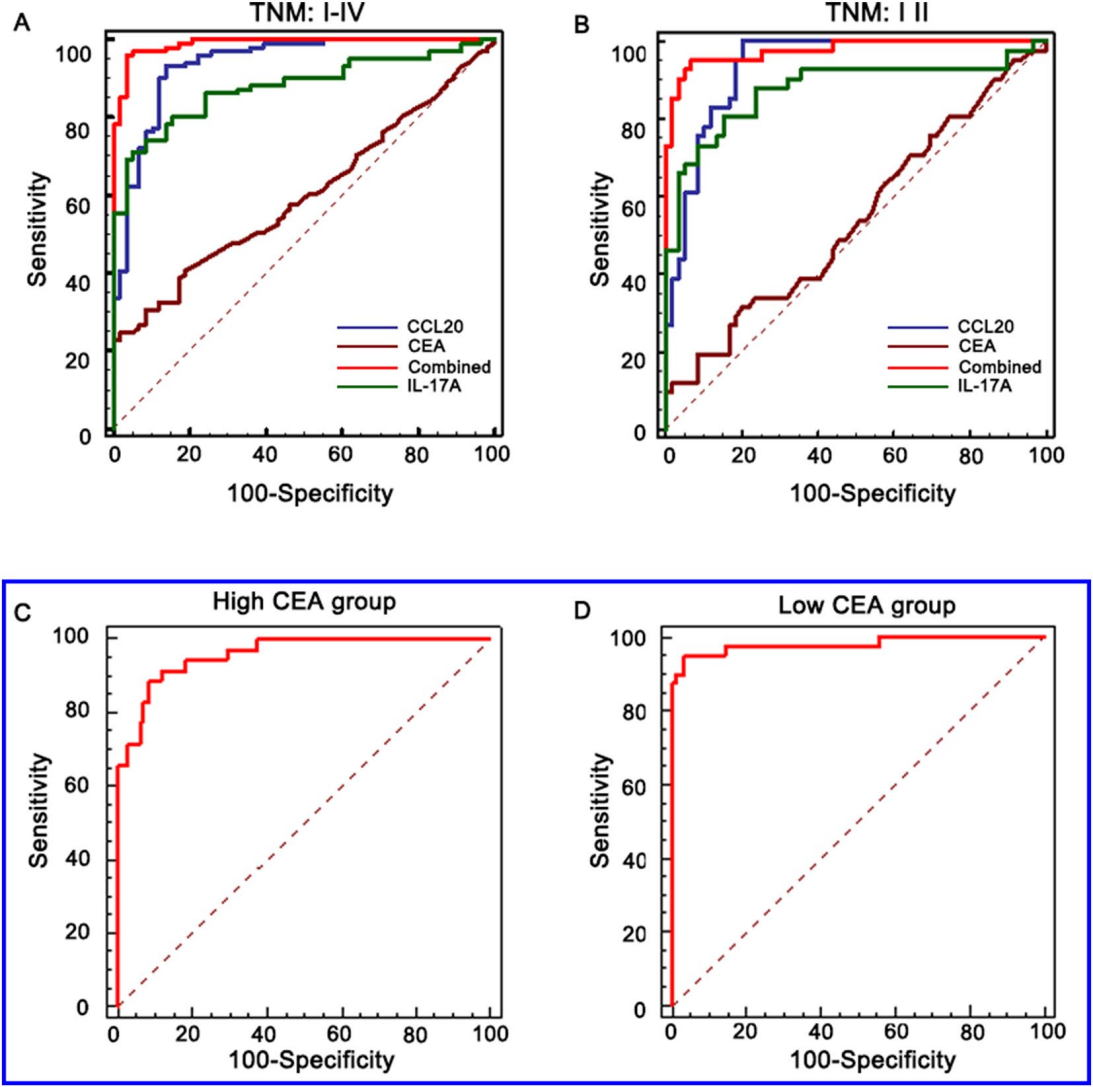
Incorrect version of Figure 4

**Fig. 2:**
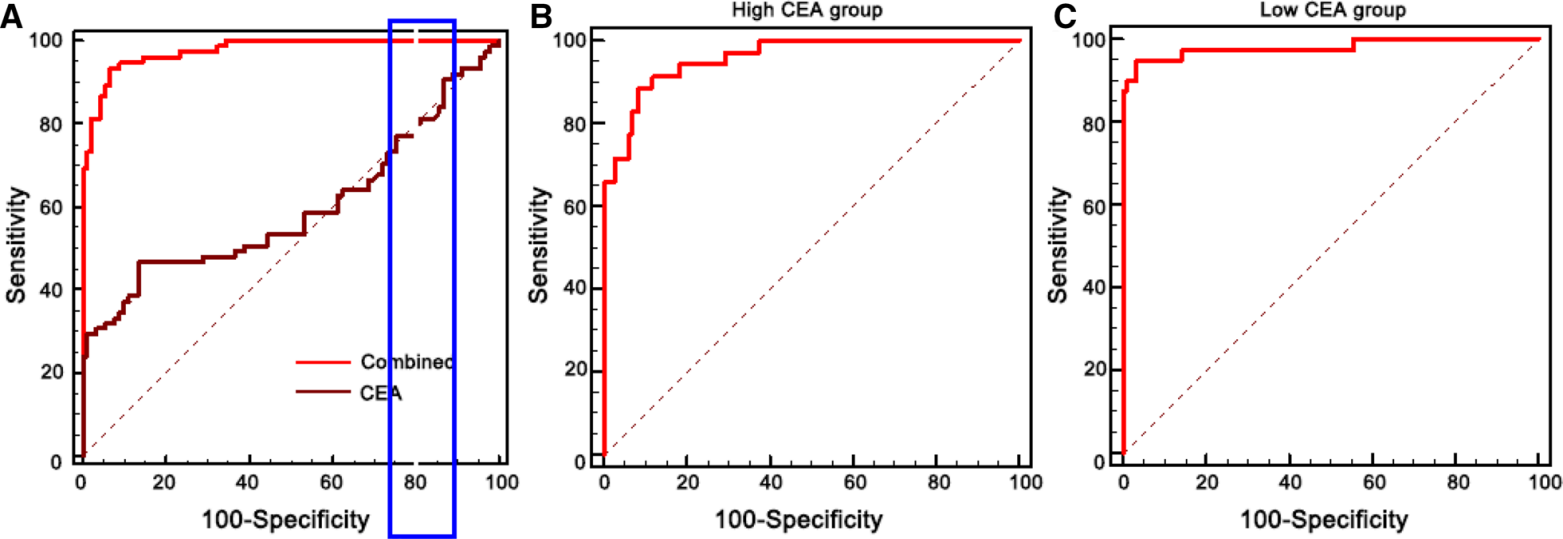
Incorrect version of Figure 5

**Fig. 3:**
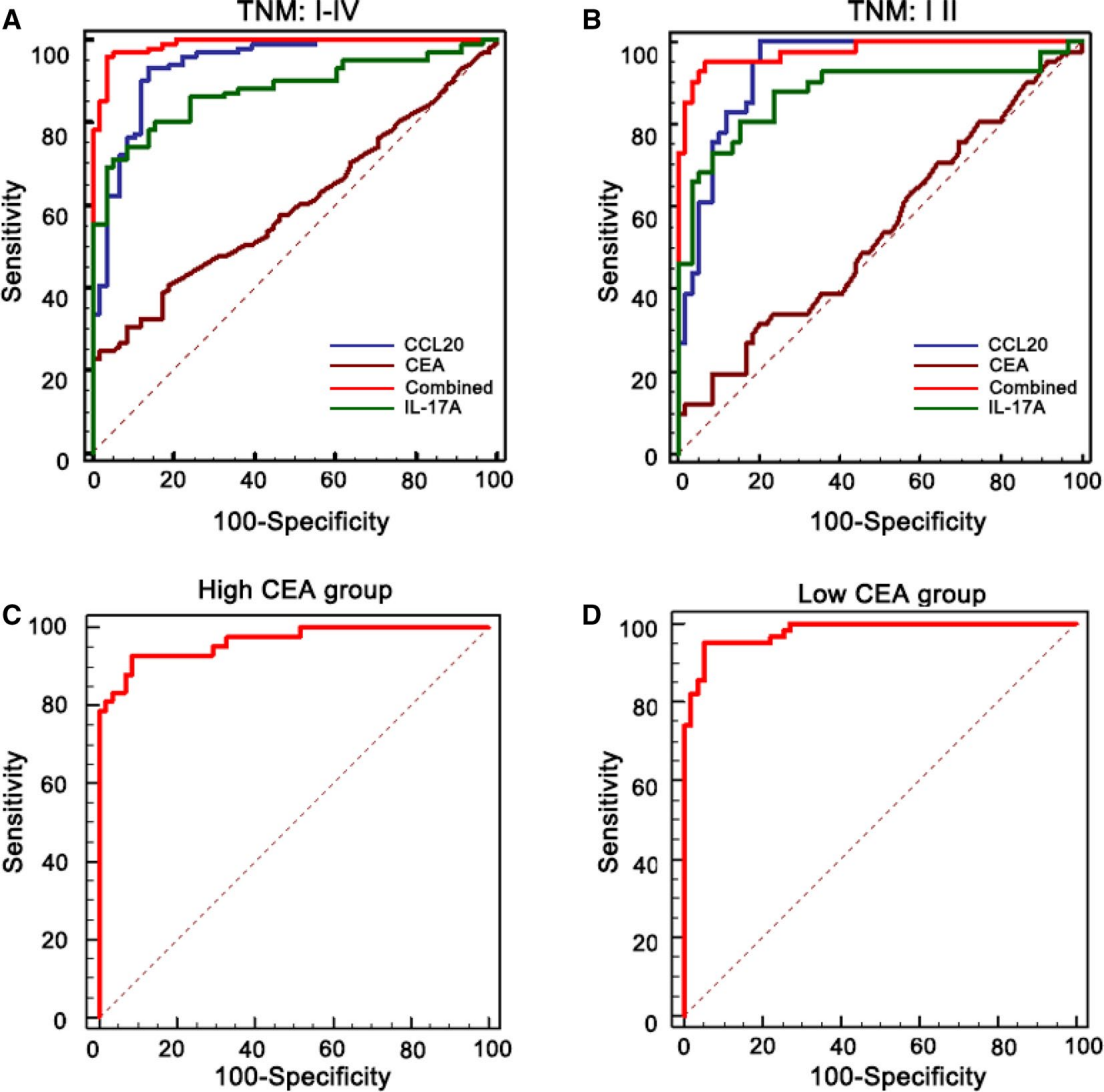
Correct version of Figure 4

**Fig. 4:**
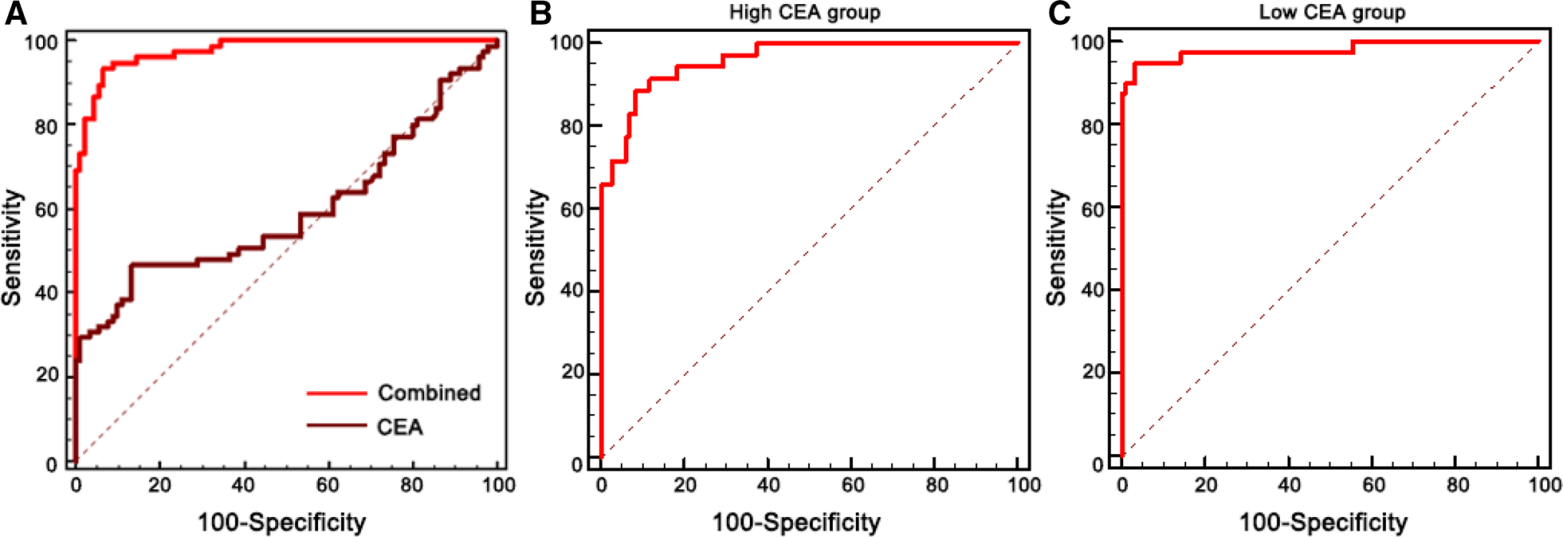
Correct version of Figure 5
