# Supplementary material for: Serum CCL20 combined with IL-17A as early diagnostic and prognostic biomarkers for human colorectal cancer
Source: J Transl Med. 2019 Aug 6;17:253. doi: 10.1186/s12967-019-2008-y (PMC6685266; doi:10.1186/s12967-019-2008-y)
Supplement: Supplementary file 1 — Additional file 1. Additional tables. [file 12967_2019_2008_MOESM1_ESM.docx]

**Table S1. Clinicopathological data for CRC patients and controls.**

| **Characteristics** | **Training phase** | **Validation phase** |
| --- | --- | --- |
| **HD** |  |  |
| **Number** | 59 | 35 |
| **Age**(mean±s.d),years | 57.6±10.1 | 54.5±11.1 |
| **Sex**, n (%) |  |  |
| Male | 31(52.5) | 15(42.9) |
| Female | 28(47.5) | 20(57.1) |
| **Colitis** |  |  |
| **Number** | 52 | 20 |
| **Age,** (mean±s.d),years | 56.4±8.2 | 55.2±10.1 |
| **Sex**, n (%) |  |  |
| Male | 28(53.8) | 11(55.0) |
| Female | 24(46.2) | 9(45.0) |
| **Type of Colitis**, n (%) |  |  |
| Crohndisease  Ulcerative gravis  Others | 10(19.2)  38(73.1)  4(7.7) | 3(15.0)  14(70.0)  3(15.0) |
| **Colorectal adenoma** |  |  |
| **Number** | 40 | 35 |
| **Age**(mean±sd),years | 56.1±12.7 | 55.2±9.2 |
| **Sex**, n (%) |  |  |
| Male | 20(50.0) | 17(48.6) |
| Female | 20(50.0) | 18(51.4) |
| **Location**, n (%) |  |  |
| Colon | 25(62.5) | 21(60.0) |
| Rectum | 15(37.5) | 14(40.0) |
| **Adenoma grade**, n (%) |  |  |
| Low-grade | 28(70.0) | 23(65.7) |
| High-grade | 12(30.0) | 12(34.3) |
| **Colorectal cancer** |  |  |
| **Number** | 112 | 75 |
| **Age**, (mean±s.d),years | 59.9±11.0 | 54.1±8.2 |
| **Sex**, n (%) |  |  |
| Male | 60(53.6) | 39(52.0) |
| Female | 52(46.4) | 36(48.0) |
| **Location**, n (%) |  |  |
| Colon  Rectum | 70(62.5)  42(37.5) | 47(62.7)  28(37.3) |
| **Tumour size**, n (%) |  |  |
| <=4cm  >4cm | 28(25.0)  84(75.0) | 19(25.3)  56(74.7) |
| **Differentiation**, n (%) |  |  |
| Well  Poor | 81(72.3)  31(27.7) | 54(72.0)  21(28.0) |
| **T stage**, n (%) |  |  |
| T1-T2 | 25(22.3) | 17(22.7) |
| T3-T4 | 87(77.7) | 58(77.3) |
| **N stage**, n (%) |  |  |
| N0 | 54(48.2) | 38(50.7) |
| N1/N2/N3 | 58(51.8) | 37(49.3) |
| **M stage**, n (%) |  |  |
| M0 | 69(61.6) | 45(60.0) |
| M1 | 43(38.4) | 30(40.0) |
| **Liver metastasis**, n (%) |  |  |
| No | 78(69.6) | 53(70.7) |
| Yes | 34(30.4) | 22(29.3) |
| **TNM stage**, n (%) |  |  |
| Stage I | 24(21.4) | 15(20.0) |
| Stage II | 35(31.2) | 23(30.7) |
| Stage III | 25(22.3) | 18(24.0) |
| StageIV | 18(16.1) | 19(25.3) |
| **Invasion**, n (%) |  |  |
| Surface | 28(25.0) | 34(45.3) |
| Deep | 84(75.0) | 41(64.7) |
| **CEA**, n (%) |  |  |
| Normal | 69(61.6) | 47(62.7) |
| High | 43(38.4) | 28(37.3) |
| **CA 19-9**, n (%) |  |  |
| Normal | 90(80.4) | 57(76.0) |
| High | 22(19.6) | 18(24.0) |

| **Table S2. Association between serum IL-17A and CCL20 levels, and clinicopathological parameters of CRC patients.** | | | | | | | | | | |  |
| --- | --- | --- | --- | --- | --- | --- | --- | --- | --- | --- | --- |
|  | | **Total** | **IL-17A** | | **X^2^** | **p Value** | **CCL20 expression** | | **X^2^** | **p Value** | |
|  | |  | **High** | **Low** |  |  | **High** | **Low** |  |  | |
| **Gender** | |  |  |  | 0.00 | 0.97 |  |  | 9.48 | **0.002*** | |
| Male | | 60 | 24 | 36 |  |  | 31 | 29 |  |  | |
| Female | | 52 | 21 | 31 |  |  | 19 | 33 |  |  | |
| **Age** | |  |  |  | 0.08 | 0.78 |  |  | 0.14 | 0.711 | |
| <60 | | 48 | 20 | 28 |  |  | 20 | 28 |  |  | |
| >=60 | | 64 | 25 | 39 |  |  | 33 | 31 |  |  | |
| **Site of lesion** | |  |  |  | 1.55 | 0.21 |  |  | 2.22 | 0.136 | |
| Rectum | | 42 | 20 | 22 |  |  | 24 | 18 |  |  | |
| Colon | | 70 | 25 | 45 |  |  | 26 | 44 |  |  | |
| **Tumor size** |  | |  |  | 0.010 | 0.91 |  |  | 4.47 | **0.04*** | |
| <4cm | | 28 | 11 | 17 |  |  | 16 | 12 |  |  | |
| >=4cm | | 84 | 34 | 50 |  |  | 34 | 50 |  |  | |
| **Pathological type** | |  |  |  | 20.97 | **<0.00***** |  |  | 10.36 | **0.00***** | |
| Adenocarcinoma | | 101 | 41 | 60 |  |  | 49 | 52 |  |  | |
| Others | | 11 | 4 | 7 |  |  | 1 | 10 |  |  | |
| **T stage** | |  |  |  | 3.85 | **0.04*** |  |  | 6.14 | **0.01*** | |
| T1-T2 | | 25 | 5 | 20 |  |  | 5 | 20 |  |  | |
| T3-T4 | | 87 | 57 | 30 |  |  | 45 | 42 |  |  | |
| **N stage** | |  |  |  | 10.27 | **0.00**** |  |  | 4.67 | **0.03*** | |
| N0 | | 54 | 20 | 34 |  |  | 22 | 32 |  |  | |
| N1/N2/N3 | | 58 | 35 | 13 |  |  | 28 | 30 |  |  | |
| **M stage** | |  |  |  | 10.57 | **0.00**** |  |  | 9.96 | **0.00**** | |
| M0 | | 69 | 23 | 46 |  |  | 23 | 46 |  |  | |
| M1 | | 43 | 22 | 21 |  |  | 27 | 16 |  |  | |
| **Liver metastasis** | |  |  |  | 4.47 | **0.03*** |  |  | 15.87 | **<0.00***** | |
| No | | 78 | 28 | 50 |  |  | 26 | 52 |  |  | |
| Yes | | 34 | 17 | 17 |  |  | 34 | 10 |  |  | |
| **Stage number** | |  |  |  | 3.77 | **0.04*** |  |  | 8.35 | **0.00**** | |
| I/II | | 59 | 26 | 33 |  |  | 25 | 34 |  |  | |
| III/IV | | 43 | 29 | 14 |  |  | 28 | 15 |  |  | |
| **Pathology** | |  |  |  | 1.30 | 0.25 |  |  | 1.31 | 0.25 | |
| Poor | | 31 | 9 | 22 |  |  | 11 | 20 |  |  | |
| Well | | 81 | 36 | 45 |  |  | 39 | 42 |  |  | |
| **Invasion** | |  |  |  | 4.65 | **0.03*** |  |  | 4.79 | **0.03*** | |
| Surface | | 28 | 6 | 22 |  |  | 7 | 21 |  |  | |
| Deep | | 84 | 39 | 45 |  |  | 43 | 41 |  |  | |
| **CEA** | |  |  |  | 6.32 | **0.01*** |  |  | 3.98 | **0.04*** | |
| Normal | | 69 | 27 | 42 |  |  | 33 | 36 |  |  | |
| High | | 43 | 18 | 25 |  |  | 14 | 29 |  |  | |
| **CA 19-9** | |  |  |  | 0.01 | 0.94 |  |  | 0.10 | 0.76 | |
| Normal | | 90 | 36 | 54 |  |  | 42 | 48 |  |  | |
| High | | 22 | 9 | 13 |  |  | 8 | 14 |  |  | |

*p<0.05 **p <0.01 ***p<0.001

| **Table S3. Association between serum level of IL-17A plus CCL20 together and clinicopathological parameters of CRC patients.** | | | | | | | |
| --- | --- | --- | --- | --- | --- | --- | --- |
|  | | **Total** | **Combined** | | **X^2^** | **p Value** |  |
|  | |  | **High** | **Low** |  |  |  |
| **Gender** | |  |  |  | 0.67 | 0.70 |  |
| Male | | 60 | 23 | 37 |  |  |  |
| Female | | 52 | 22 | 30 |  |  |  |
| **Age** | |  |  |  | 0.37 | 0.43 |  |
| <60 | | 48 | 22 | 26 |  |  |  |
| >=60 | | 64 | 27 | 37 |  |  |  |
| **Site of lesion** | |  |  |  | 0.24 | 0.25 |  |
| Rectum | | 42 | 21 | 21 |  |  |  |
| Colon | | 70 | 27 | 43 |  |  |  |
| **Tumor size** |  | |  |  | 0.41 | 0.49 |  |
| <4 | | 28 | 12 | 16 |  |  |  |
| >=4 | | 84 | 32 | 52 |  |  |  |
| **Pathological type** | |  |  |  | 16.96 | **<0.00***** |  |
| Adenocarcinoma | | 101 | 42 | 59 |  |  |  |
| Others | | 11 | 3 | 8 |  |  |  |
| **T stage** | |  |  |  | 18.13 | **<0.00***** |  |
| T1-T2 | | 25 | 5 | 20 |  |  |  |
| T3-T4 | | 87 | 59 | 28 |  |  |  |
| **N stage** | |  |  |  | 19.94 | **<0.00***** |  |
| N0 | | 54 | 19 | 35 |  |  |  |
| N1/N2/N3 | | 58 | 38 | 10 |  |  |  |
| **M stage** | |  |  |  | 11.91 | **0.001**** |  |
| M0 | | 69 | 24 | 45 |  |  |  |
| M1 | | 43 | 23 | 20 |  |  |  |
| **Liver metastasis** | |  |  |  | 4.96 | **0.03*** |  |
| No | | 78 | 22 | 56 |  |  |  |
| Yes | | 34 | 17 | 17 |  |  |  |
| **Stage number** | |  |  |  | 7.51 | **0.006**** |  |
| I/II | | 59 | 25 | 34 |  |  |  |
| III/IV | | 43 | 30 | 13 |  |  |  |
| **Pathology** | |  |  |  | 0.35 | 0.55 |  |
| Poor | | 31 | 10 | 21 |  |  |  |
| Well | | 81 | 31 | 50 |  |  |  |
| **Invasion** | |  |  |  | 4.54 | **0.03*** |  |
| Surface | | 28 | 6 | 22 |  |  |  |
| Deep | | 84 | 37 | 47 |  |  |  |
| **CEA** | |  |  |  | 7.10 | **0.008**** |  |
| Normal | | 69 | 21 | 48 |  |  |  |
| High | | 43 | 19 | 24 |  |  |  |
| **CA 19-9** | |  |  |  | 0.27 | 0.60 |  |
| Normal | | 90 | 34 | 56 |  |  |  |
| High | | 22 | 7 | 15 |  |  |  |

***p<0.05 **p <0.01 ***p<0.001**

**Table S4. Cox’s proportional hazard model analysis of prognostic factors in CRC patients.**

|  | **Univariate** | | |  | **Multivariate** | | |
| --- | --- | --- | --- | --- | --- | --- | --- |
|  | **HR** | **95%CI** | **p Value** |  | **HR** | **95%CI** | **p Value** |
| **Sex**  (Female vs Male) | 1.14 | 0.58-2.25 | 0.70 |  |  |  |  |
| **Tumor size**  (>=4cm vs <4cm) | 3.69 | 0.31-1.51 | 0.35 |  |  |  |  |
| **Pathological type** (Adenocarcinoma vs Others) | 1.14 | 0.38-3.43 | 0.81 |  |  |  |  |
| **T stage**  (T3-T4 vs T1-T2) | 2.14 | 0.99-4.61 | 0.05 |  |  |  |  |
| **Pathology**  (Well vs poor) | 1.48 | 1.36-3.85 | 0.39 |  |  |  |  |
| **Invasion**  (Deep vs Surface) | 1.77 | 0.79-3.56 | 0.14 |  |  |  |  |
| **CEA**  (High vs Normal) | 1.16 | 0.58-2.32 | 0.69 |  |  |  |  |
| **Liver metastasis**  (Yes vs No) | 2.32 | 2.15-5.17 | **0.04*** |  |  |  |  |
| **N**(N1/N2/N3 vs N0) | 2.60 | 1.22-5.09 | **0.00**** |  |  |  |  |
| **M**(M1 vs M0) | 1.99 | 0.99-4.65 | **0.03*** |  |  |  |  |
| **Age**  (<60 vs >=60) | 1.97 | 1.00-3.89 | **0.04*** |  | 1.98 | 0.97-1.59 | **0.04*** |
| **Site of lesion**  (Rectum vs Colon) | 1.52 | 1.49-2.50 | **0.04*** |  | 3.14 | 1.07-5.62 | **0.02*** |
| **Stage number**  (III/IV vs I/II) | 1.69 | 1.80-4.16 | **0.02*** |  | 1.37 | 1.16-3.13 | **0.03*** |
| **CCL20**  (High vs Low) | 2.33 | 1.10-4.21 | **0.02*** |  | 1.12 | 1.10-1.45 | **0.00**** |
| **IL17A**  (High vs Low) | 2.32 | 1.17-4.23 | **0.01*** |  | 1.22 | 0.68-1.15 | **0.04*** |
| **Combination** |  |  |  |  |  |  |  |
| (High vs Low) | 2.46 | 1.23-4.37 | **0.03*** |  | 1.45 | 1.01-2.31 | **0.04*** |

*p<0.05 **p <0.01 ***p<0.001
