# Supplementary material for: Serum CCL20 combined with IL-17A as early diagnostic and prognostic biomarkers for human colorectal cancer
Source: J Transl Med. 2019 Aug 6;17:253. doi: 10.1186/s12967-019-2008-y (PMC6685266; doi:10.1186/s12967-019-2008-y)
Supplement: Supplementary file 2 — Additional file 2. Additional methods. [file 12967_2019_2008_MOESM2_ESM.doc]

**Additional materials and methods:**

**ELISA**

Cytokine levels were measured by the highly sensitive enzyme-linked immune sorbent assay (ELISA) kits (R&D Systems Minneapolis, MN) specific for the human cytokines according to the manufacturer’s instruction. Briefly, the standard stocks were serially diluted in Reagent Diluent to generate 7 points for the standard curves. Diluted Capture Antibody was added to a 96-well, flat-bottomed, polystyrene microtiter plate, at final volume of 100 μl. Plates were sealed and incubated overnight at room temperature, then washed with Washing Buffer using an autowasher. Premixed standards or samples (100 μl) were added to each well containing washed beads, covered with an adhesive strip and incubated for 2 hours at room temperature. 100 μl of the premixed Detection Antibody was added to each well and the plate was covered with a new adhesive strip and incubated for 2 hours at room temperature. After incubation and washing, Streptavidin-HRP was added to each well (100 μl). The incubation was terminated after 20 min at room temperature and the plates were kept away from direct light. A substrate solution was added into each well; colour development was terminated by stop solution. The resulting absorbance was read at 450 nm using a spectrophotometer.

**Immunohistochemistry**

The protocols of immunohistochemistry were described elsewhere {Wang, 2012 #80}. The staining was performed on formalin-fixed, paraffin tissue blocks of CRC. Mouse anti-CCL20, rabbit anti-human IL-17A (1:300; Abcam, USA) were used as primary antibodies. For immunohistochemistry, 3 fields of images per sample were taken. Negative controls were treated identically, but without the primary antibody. Then, the specimens were briefly washed in PBS and incubated at room temperature with the anti-goat antibody and avidin–biotin peroxidase. Images were analyzed using a fluorescent microscope (Olympus, IX71, Japan). Assessment of immunohistochemical staining was evaluated by two independent pathologists. A final immune reactivity scores (IRS) were obtained for each case by multiplying the percentage and the intensity score.

**Gene ontology (GO) analysis**

Functional analysis of differentially expressed genes was carried out by the Gene Ontology project ([http://www.geneontology.org](http://www.geneontology.org/)) on the basis of biological process. The Fisher’s exact test and X2 test were used to classify the GO category, and the FDR was calculated to correct the p-value. P Value < 0.05 and FDR < 0.1 were used as a threshold to select significant GO categories.

**Gene set enrichment analyses (GSEA)**

GSEA was performed by the GSEA software and gene sets used in this work were downloaded from the Molecular Signatures Database (http://software. broadinstitute.org/gsea/msigdb/index.jsp, MSigDB v4.0, released Jun 7, 2013).
