# Supplementary figures and images for: Serum CCL20 combined with IL-17A as early diagnostic and prognostic biomarkers for human colorectal cancer
Source: J Transl Med. 2019 Aug 6;17:253. doi: 10.1186/s12967-019-2008-y (PMC6685266; doi:10.1186/s12967-019-2008-y)

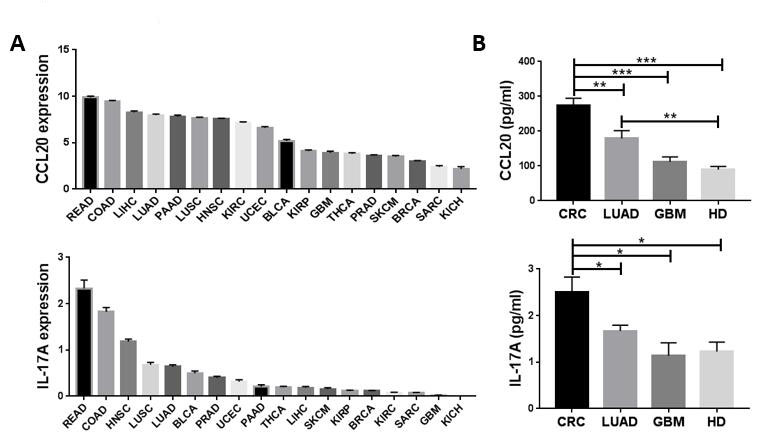

Supplement: Supplementary file 3 — Additional file 3: Fig. S1. CCL20 and IL-17A expression are higher in CRC than the expression in other cancers. A. The expression of CCL20 and IL-17A from TCGA database in 18 kinds of cancer tissues. B. The levels of CCL20 and IL-17A in sera from CRC patients, lung adenocarcinoma [LUAD] patients, glioblastoma [GBM] patients and controls (Healthy donors [HD]) were tested by Elisa (*P < 0.05; **P < 0.01; ***P < 0.001). [file 12967_2019_2008_MOESM3_ESM.jpg]
